# PKA and cAMP/CNG Channels Independently Regulate the Cholinergic Ca^2+^-Response of *Drosophila* Mushroom Body Neurons^1^,^2^,^3^

**DOI:** 10.1523/ENEURO.0054-14.2015

**Published:** 2015-04-30

**Authors:** Pierre Pavot, Elena Carbognin, Jean-René Martin

**Affiliations:** Institut des Neurosciences Paris-Saclay (Neuro-PSI), UMR-9197, CNRS/Université Paris Sud, 91198, Gif-sur-Yvette, France

**Keywords:** cAMP, CNG-channels, Drosophila, functional calcium brain imaging, genetics, nicotinic acethylcholine receptor

## Abstract

The mushroom bodies (MBs), one of the main structures in the adult insect brain, play a critical role in olfactory learning and memory. Though historical genes such as *dunce* and *rutabaga*, which regulate the level of cAMP, were identified more than 30 years ago, their *in vivo* effects on cellular and physiological mechanisms and particularly on the Ca^2+^-responses still remain largely unknown. In this work, performed in *Drosophila*, we took advantage of *in vivo* bioluminescence imaging, which allowed real-time monitoring of the entire MBs (both the calyx/cell-bodies and the lobes) simultaneously. We imaged neuronal Ca^2+^-activity continuously, over a long time period, and characterized the nicotine-evoked Ca^2+^-response. Using both genetics and pharmacological approaches to interfere with different components of the cAMP signaling pathway, we first show that the Ca^2+^-response is proportional to the levels of cAMP. Second, we reveal that an acute change in cAMP levels is sufficient to trigger a Ca^2+^-response. Third, genetic manipulation of protein kinase A (PKA), a direct effector of cAMP, suggests that cAMP also has PKA-independent effects through the cyclic nucleotide-gated Ca^2+^-channel (CNG). Finally, the disruption of calmodulin, one of the main regulators of the *rutabaga* adenylate cyclase (AC), yields different effects in the calyx/cell-bodies and in the lobes, suggesting a differential and regionalized regulation of AC. Our results provide insights into the complex Ca^2+^-response in the MBs, leading to the conclusion that cAMP modulates the Ca^2+^-responses through both PKA-dependent and -independent mechanisms, the latter through CNG-channels.

## Abstract (precis)

The mushroom bodies (MBs) are the most prominent structures in adult *Drosophila* brain. They have been involved in several crucial functions, such as learning and memory, sleep, locomotor activity, and decision making.

## Significance Statement

The mushroom bodies (MBs) are the most prominent structures in adult *Drosophila* brain. They have been involved in several crucial functions, such as learning and memory, sleep, locomotor activity, and decision making. However, although the historical genes such as *dunce* and *rutabaga*, which regulate the cAMP level, were identified more than 30 years ago, their effect on the cellular and physiological mechanisms of the Ca^2+^-response still remain largely unknown. Here, using an *in vivo* functional Ca^2+^-imaging approach, we describe the roles of the different components of the cAMP signaling pathways in the MBs. These results may serve as a foundation for disentangling the complex roles of cAMP in memory formation, as well as guiding new behavioral experiments that focus on CNG-channels and calmodulin.

## Introduction

Several behavioral and genetic studies performed in different invertebrate organisms, such as honeybees, locusts, and the fruitfly, *Drosophila melanogaster*, demonstrated the critical role of the mushroom bodies (MBs) in olfactory learning and memory (L&M) (Heisenberg, 1998; 2003; Menzel, 2012), as well as in other functions such as sleep (Joiner et al., 2006), locomotor activity (Martin et al., 1998; Besson and Martin, 2005), and decision making (Tang and Guo, 2001). In *Drosophila*, a combination of genetic and behavioral studies, based on an extensive library of mutants and transgenic animals, have identified a number of genes and signaling cascades that contribute to memory formation (Davis, 2005; McGuire et al., 2005; Keene and Waddell, 2007; Busto et al., 2010). Genes such as *dunce* (*dnc*) (Dudai et al., 1976), encoding a phosphodiesterase (PDE) that degrades cAMP, and *rutabaga* (*rut*) (Duerr and Quinn, 1982), encoding an adenylate cyclase (AC) that synthesizes cAMP, are known to regulate cAMP levels. Although these genes were identified more than 30 years ago, their precise roles and physiological consequence of disrupting cAMP levels is largely unknown. Furthermore, the Ca^2+^-response that contributes to several cellular processes and even gene expression, yielding *in vivo* memory formation in the MBs, is still not well characterized. This lack of information is mainly due to the limited electrophysiological access to the neurons in the brain of adult *Drosophila*, though in bigger invertebrates such as honeybee, some electrophysiological studies have been performed (Schäfer et al., 1994). Indeed, the majority of studies, notably on *dnc* and *rut* mutants, have been performed on other neurons, such as motoneurons (Ueda and Wu, 2009) or on dissected brains, at different developmental stages (Lee and O’Dowd, 2000).

More recently, physiological approaches using fluorescent markers to image the fly brain have begun to explore MB physiology. Except in the few studies that have investigated both the calyx and the lobes (Tomchik and Davis, 2009), up to now the majority of calcium imaging studies were focused on single MB regions, either on a part of the calyx/cells bodies (Wang et al., 2001; 2004; 2008; Honegger et al., 2011) or on the lobes (Yu et al., 2006; Akalal et al., 2010). Thus, in adult flies, except for protein kinase A (PKA) quantification (Gervasi et al., 2010), the direct *in vivo* effect of disturbing the cAMP signaling pathway on the Ca^2+^-response and on the overall cellular physiology of the Kenyon cells (KCs) still remains poorly characterized.

In this work, we took advantage of the *in vivo* bioluminescence imaging technique recently developed (Martin et al., 2007) to simultaneously monitor neuronal Ca^2+^-activity of the whole MB structure, including the calyx/cell-bodies (CCB) and the lobes, continuously, over a long time period. We recorded the nicotine (cholinergic)-induced Ca^2+^-response, employing both genetics (using mutants and/or targeted RNAi) and pharmacological approaches to manipulate different components of the cAMP signaling pathway. We show that the downregulation or upregulation of cAMP levels results in a proportional change of the Ca^2+^-response, while acute increase in the cAMP levels is sufficient to trigger a Ca^2+^-response. Finally, genetic manipulation of PKA, a cAMP effector, suggests that cAMP also has a PKA-independent effect, via the cyclic nucleotide-gated Ca^2+^-channel (CNG).

## Materials and Methods

### Flies

Flies were maintained on standard medium at room temperature (24 °C). P[UAS-GFP-aequorin] (GA) transgenic flies (Martin et al., 2007) were used in conjunction with the P[GAL4]OK107 line to target GA to the MBs. P[GAL4]OK107 (Bloomington Stock Center) is expressed in a large population (approximately 90%) of KCs (Aso et al., 2010). Imaging experiments were performed on progeny of flies containing both the P[GAL4]OK107 driver and the P[UAS-GA] transgene (GA/CS;OK107/CS) (CS = Canton-S) in transheterozygotes. We used specific RNAi: (P[UAS-*rutabaga-*RNAi]: *rut*-RNAi(1)=VDRC-101759-KK, *rut*-RNAi(2)=VDRC-5569-GD, P[UAS-*dunce-*RNAi]: *dnc*-RNAi(1)=VDRC-107967-KK, *dnc*-RNAi(2)=NIG, P[UAS-*CaM-*RNAi]: cam-RNAi(1)=VDRC-102004-KK, cam-RNAi(2)= VDRC-28242-GD, P[UAS-*cngc-*RNAi]: *cngc*-RNAi(1)=VDRC-101745-KK, *cngc*-RNAi(2)= VDRC-28625-GD, P[UAS-*cngl-*RNAi]: *cngl*-RNAi(1)=VDRC-102411-KK, *cngl*-RNAi(2)=VDRC-40964-GD, from two different collections from Vienna Drosophila Resource Center (VDRC) and from R. Ueda (National Institute of Genetics (NIG), Mishima, Shizuoka, Japan) to knock-down the genes investigated specifically in the MBs. We use the P[UAS-G_αs_*] provided by C. O’Kane (Department of Genetics, University of Cambridge, Cambridge, UK) to activate the *rut*-AC. We overexpressed *rut* and *dnc* specifically in the MBs using the transgenic constructs *UAS-rut* (Zars et al., 2000) and *UAS-dnc* (Cheung et al., 1999) provided by G. Isabel (Université Paul Sabatier Toulouse III, Toulouse, France) and T. Preat (ESPCI, Paris, France), respectively. We overexpressed the P[UAS-R*], a mutated PKA regulatory subunit to block the PKA, and the P[UAS-mC*] to permanently mimic the activation of the PKA, and consequently its target. Both lines were provided by D. Kalderon (Columbia University, Biological Sciences, New York, NY). As controls, we tested VDRC control-RNAi genetic background lines, both the KK series (VDRC-61000: control RNAi-1) and the GD series (VDRC-60000: control RNAi-2) in heterozygotes. Moreover, the exchange of the genetic background of the line (GA/CS;OK107/CS) (cantonized) for a “Berlin” genetic background (GA/Ber;OK107/Ber) (Berlinized) did not modify the level of the Ca^2+^-response (data not shown). We therefore use the UAS-RNAi lines in trans-heterozygotes (GA;OK107xUAS-RNAi=GA/UAS-RNAi;OK107/+). All experiments were performed on females.

### Brain preparation

Preparation of flies for live *in vivo* brain imaging was performed as described Martin et al. (2007). In brief, a 4-d-old female fly was briefly cold (ice) anesthetized, inserted in a truncated 1 ml commercial pipette tip until the head protruded and was fixed and sealed in place with biology-compliant dental glue (Protemp IV, ESPE). The assembly was then placed in the back of a recording chamber and secured with silicone glue (ESPE). The recording chamber (1 ml) was filled with Ringer’s solution (Martin et al., 2007) and a tiny window in the head capsule was cut out to expose the MBs. Care was taken not to damage the brain. In order to weaken and permeabilize the neuro-epithelium to allow better drug diffusion and coelenterazine (the GFP-aequorine cofactor) penetration, the opened heads were incubated at room temperature in Ringer’s solution containing 10 U/ml papain (Sigma) activated by 5 mM L-cysteine (Sigma) for 10 min (Gu and O’Dowd, 2006). Brains were washed four times with Ringer solution, then incubated in *Drosophila* Ringer’s solution containing 5 µM benzyl-coelenterazine (NanoLight, Prolume) for 2 h before experiments.

### *In vivo* brain imaging

Nicotine-induced Ca^2+^-response (bioluminescence signals) in the MBs were monitored with an electron multiplier CCD camera (EM-CCD, Andor, iXon; cooled to −80 °C) fitted onto a microscope (Eclipse-E800, Nikon). The setup was housed inside a tight dark box (Sciences Wares) to avoid any undesired (ambient) light contamination. We used a 20× immersion-objective lens (NA 0.5, Plan Fluor, Nikon), giving a field of view of 400 × 400 µm (512 × 512 pixels). To improve signal-to-noise ratio, data were acquired with a 0.25 s integration time (4 Hz), and 2 × 2 binning was used (1 pixel = 1.2 × 1.2 μm). To acquire and store data, each detected photon was assigned *x*,*y*-coordinates and a time point.

### Perfusion system

All drug applications were controlled externally using a six-way multivalves gravity perfusion system (VC 6 Standard, Warner). The flow was controlled using six volumetric perfusion regulators (Dosi-flow 3, Leventon) calibrated prior each recording session for a flow of 2 ml/min. Simultaneously, 2 ml/min of liquid were extracted from the recording chamber using a peristaltic pump (Minipuls 2, Gilson) to allow a continuous flow. All tubing was bio-compliant (Tygon R3603, St-Gobain).

### Pharmacology

To stimulate the flies, we used either acetylcholine or nicotine. To investigate the roles of cAMP pathway, forskolin and IBMX were used. Nicotine (Sigma) was prepared as a 10 mM stock solution in H_2_O and diluted to 25 μM in *Drosophila* Ringers just prior experiment. Forskolin (Sigma) was prepared as a 13 mM stock in ethanol and then dissolved in *Drosophila* Ringers to 13 µM. IBMX (Sigma) was daily dissolved at 40 mM in 100% ethanol and diluted further in *Drosophila* Ringers at 200 µM final concentration. 8Br-cAMP (Sigma) was dissolved at 20 mM in 100% ethanol and diluted further in *Drosophila* Ringers at 200 µM final concentration. All drugs were applied using the previously described perfusion system.

### Determination of the nicotine-induced Ca^2+^-response

The duration and the total photons (TP) were determined using an automated statistical analysis of the signal script developed at the laboratory (a routine programmed in Microsoft Visual Basic/Excel, available on request). Briefly, a sliding window of 20 data points (5 s) was compared using *t* test with a control window of 240 data points (30 s) corresponding to the recorded resting phase before nicotine application. The response of the KCs was considered (quantified) between the time we obtained more than six of 10 consecutive *p* values above 0.025 (starting response) and more than six of 10 consecutive *p* values below 0.025 (end of response).

### Quantitative and statistical analysis

We used the Photon Viewer (2.1) software (Science Wares) written in LabView 7.1 (National Instruments) to analyze the imaging data. Nicotine-induced bioluminescence signals are presented as photons/s (within the ROI). Image recordings were obtained from five to 15 flies for each genotype. All statistics were done using InVivoStat (2.1) software (Clark et al., 2012), a biostatistics front-end for the open-source statistic package based on the R project (http://www.r-project.org/). The dataset was analyzed via one-way ANOVA followed by a planned comparison on the predicted means to compare the level of the selected effect using the Benjamini-Hochberg's with a rank transformation (Benjamini and Hochberg, 1995; Benjamini et al., 2001).

## Results

In *Drosophila*, as in mammals, the olfactory integration network is composed of at least two successive integration nodes (synapses) linked by nerve bundles. The odor, transduced by the olfactory receptors neurons (ORNs), is first integrated in the antennal lobe glomeruli (Wilson and Mainen, 2006; Wilson, 2013). This integration occurs through a complex network of local interneurons and dendrodendritic connections with the projection neurons (PNs) (Ng et al., 2002; for review, see Wilson, 2013). The PNs send their axons to two distinct structures: the calyx of the MBs (which represent the dendritic arborisation of KCs) and the lateral horn. Synaptic connections between PNs and the KCs are the second critical site of integration of the olfactory input (Murthy et al., 2008; Turner et al., 2008; Cassenaer and Laurent, 2012). These excitatory PN−KC synapses are cholinergic, with KCs expressing ionotropic nicotinic acethylcholine receptor (nAchR) (Fayyazuddin et al., 2006). In this study, we focused on intrinsic KC physiology, a part of the network described above. In order to stimulate nAChRs on the MBs and trigger Ca^2+^-responses, we first applied the endogenous agonist acetylcholine (Ach), which triggers a Ca^2+^-response when applied to dissected brains, as reported previously (Yu et al., 2003). However, Ach is unstable and can potentially affect other receptor types, like the muscarinic Ach receptor, located in other parts of the olfactory integration network, such as the antennal lobes (Blake et al., 1993). Hence, we use nicotine, which is more stable and allows better and more reliable stimulus control. As previously reported in pupae MB cultures (Campusano et al., 2007), nicotine application evokes a similar response pattern as Ach application, but is more reproducible.

### Nicotine induced a characteristic Ca^2+^-response in mushroom bodies

**Figure 1:**
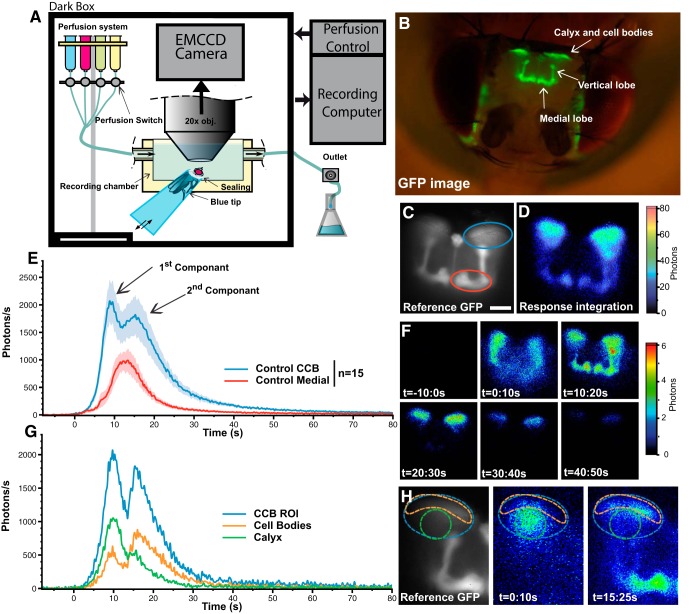
Schematic view of the setup and a representative nicotine-evoked Ca^2+^-response in the MBs of a control fly. ***A,*** Recording setup. The head capsule of a living fly is opened and the brain is bathed in Ringer’s solution, into which the agonist or antagonist is applied. ***B,*** Fluorescent image, taken with a Dim+Fluorescent light, of a 4-d-old female control fly (GFP-aequorin/CS; OK107/CS) after preparation and dissection. ***C,*** Fluorescent image of the MBs taken at the beginning of the experiment and used as the reference image. Light emission was quantified from the blue and red circles, which represent the CCB and the ML ROIs, respectively (scale bar, 50 μm). ***D,*** Bioluminescence image (accumulation time: 120 s) of the nicotine-evoked response in a typical control fly. ***E,*** Bioluminescent Ca^2+^-activity profile in MBs, evoked by nicotine (*n* = 15). Values are mean ± SEM. ***F,*** Six sequential bioluminescence images from *t* = −10 s to *t* = 50 s (accumulation time: 10 s) of the nicotine-evoked response. ***G,*** Decomposition image of the CCB showing that the first component corresponds to the response in the calyx (dendrites, ROI circled in green in ***H***), while the second corresponds to that of the cell-bodies (ROI circled in orange in ***H***). Because of the recording angle, the response in the calyx, unavoidably, partially overlaps with the response in the cell bodies. ***H,*** Accumulated (10 s) bioluminescence image of the nicotine-evoked response corresponding to each ROI, separately. Because it is not possible to perfectly separate the response from the two ROIs, we use a single ROI comprised of both of them: the CCB complex (ROI circled in blue). For the medial lobes (red circle in ***C***), again here, since we privileged the overall view of the MBs, this approach did not permit us to separate the response of the various sublobes, such as β, β’, or γ.

We used a 20× objective, which allowed visualisation of the entire MB at once, and recorded responses from the CCB and various MB lobes (Fig. 1*A−C*), which could be subdivided into the vertical lobe, comprised of the α/α’ lobes, and the medial lobes, comprised of the β/β’ and γ lobes. In the absence of any stimulus, we observed neither basal nor oscillatory Ca^2+^-activity in the KCs (the constitutive neurons of the MBs). A 1 min application of nicotine (25 μM, at 2 ml/min) evoked a typical response pattern in the MBs. The response started in the CCB and propagated into the axonal projections at the level of the MB lobes (Fig. 1*D−F*). A typical nicotine-evoked Ca^2+^-response was composed of two distinct phases in the CCB, and only one phase in the MB lobes. The CCB response first showed a rapid exponential activity increase (0 s corresponds to the beginning of the response), and peaked at approximately 9 s (Fig. 1*E*). This first phase reached ∼2200 photons/s (ph/s), the signal then decreased slightly for ∼2 s, and rose again to give a second lower peak of ∼1800 ph/s, ∼15 s after the first response started. The responses finally decreased slowly, and terminated after ∼80 s. To simplify, the response can be summarized into two components, which are defined by the first and the second peak. In addition, the use of different angles of view to observe the MB permitted the identification of substructures associated with both response components. The first component corresponds to the response in the calyx (Fig. 1*H*, green ROI), while the second component, which occurs slightly after, corresponds the response in the cell bodies of the KCs (Fig. 1*H*, orange ROI). Indeed, a refinement of the two ROIs, which was possible on few flies according to their precise angles of view, allowed spatiotemporal separation of these two components of the response (Fig. 1*G*). However, as the two components partly overlap in the majority of the flies imaged, it made it difficult to precisely and systematically separate the two components and to define their individual durations. Consequently, only the overall response of the CCB and duration were taken into account in this study.

**Figure 2:**
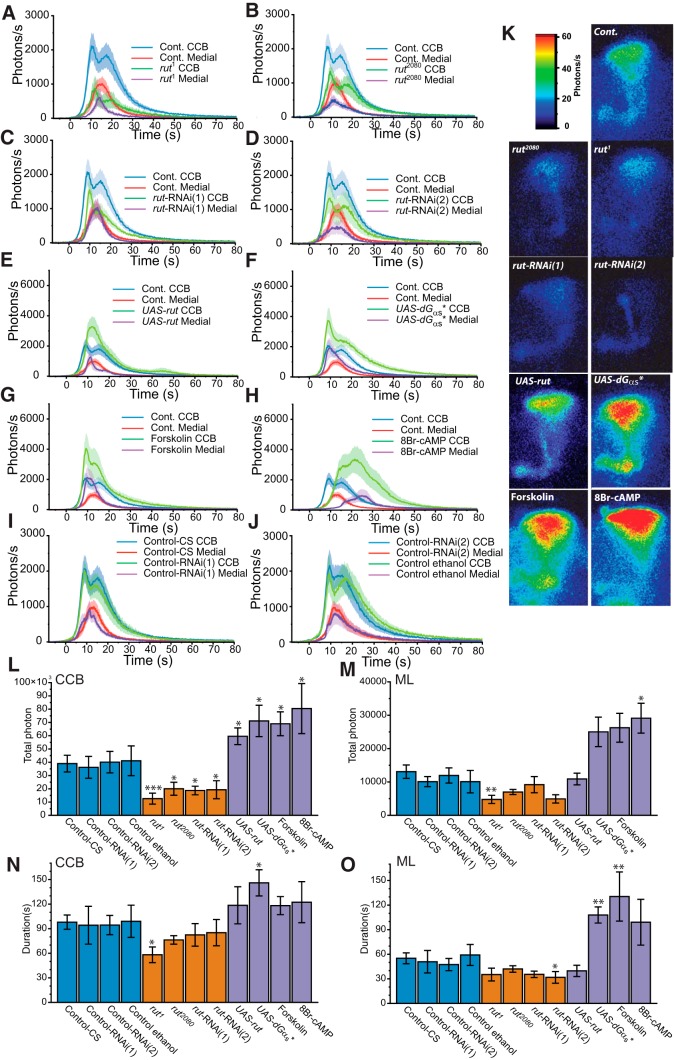
Modulation of nicotine-evoked transient Ca^2+^-response related to the cAMP pathway through *rut*. ***A−D***, Bioluminescent Ca^2+^-activity profile in MBs evoked by nicotine with downregulated cAMP production in *rut^1^* (*n* = 6), *rut^2080^* (*continued in page 7*). (*n* = 7), *rut*-RNAi(1) (*n* = 8), and *rut*-RNAi(2) (*n* = 6). ***E−H***, Bioluminescent Ca^2+^-activity evoked by nicotine with upregulated cAMP production in *UAS-rut* (*n* = 8), UAS-G_αs_* (*n* = 7), flies incubated 10 min with forskolin (13 μM; *n* = 6), and flies incubated 10 min with 8Br-cAMP (200 μM; *n* = 7). ***I***, ***J***, Bioluminescent nicotine-evoked Ca^2+^-activity with different genetic background controls in Control-RNAi(1) (VDRC-60100: *n* = 17; ***I***) and in Control-RNAi(2) (VDRC-60000: *n* = 15; ***J***) as well as in Control-RNAi(2), incubated 10 min with ethanol 1/200 (*n* = 11; ***J***). Values are mean ± SEM. ***K***, Bioluminescent image (accumulation time: 120 s) of the nicotinic Ca^2+^-response of a typical fly for each genotype (except for the other controls presented in ***I*** and ***J***). ***L***, ***M***, Total number of photons during the nicotine response in the CCB (***L***) and in the ML (***M***). ***N***, ***O***, Total duration of the response in the CCB (***N***) and in the medial lobe (***O***). Values are mean ± SEM. Statistics: ***A−D***, One-way ANOVA was followed by a planned comparison of the predicted means to compare the levels of the selected effect using the Benjamini-Hochberg's test with rank transformation: *p < 0.05; **p < 0.01; ***p < 0.001 (for complete statistics, see Tables 1 and 2).

**Figure 3:**
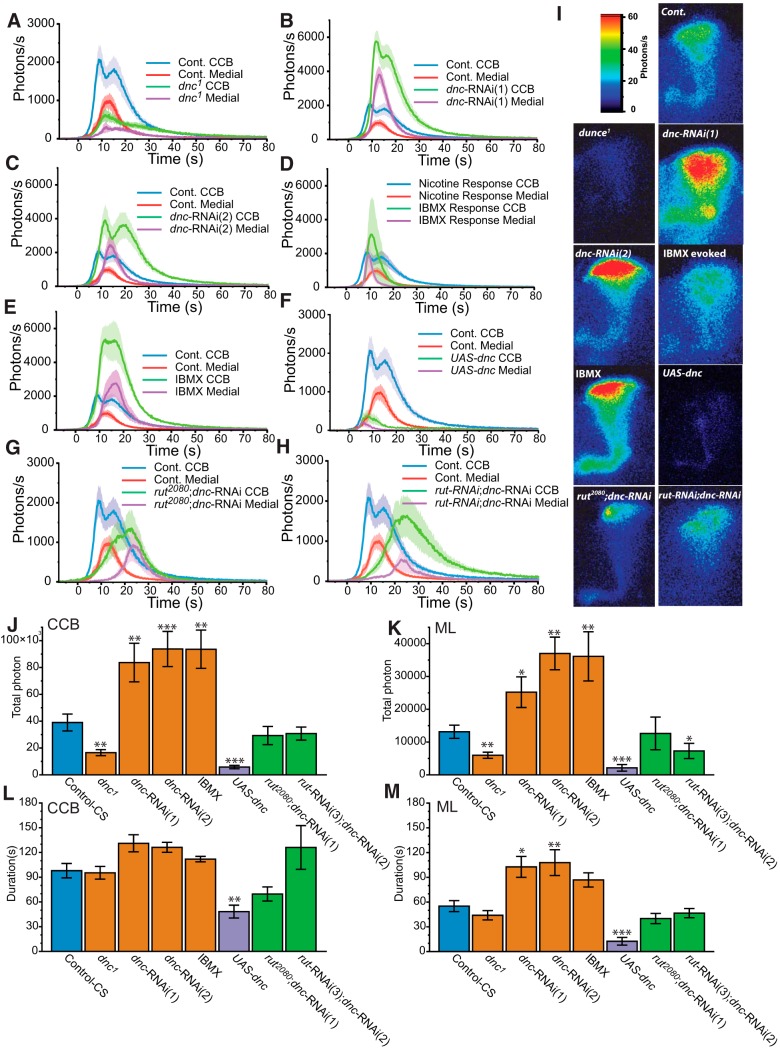
Modulation of nicotine-evoked transient Ca^2+^-response related to the cAMP pathway through *dnc*. ***A−E***, Bioluminescent Ca^2+^-activity with downregulated degradation of cAMP through a perturbation of the *dnc*-PDE. ***A−C***, *dnc^1^* mutant flies (*n* = 14, ***A***), *dnc-*RNAi(1) (*n* = 9, ***B***), and *dnc*-RNAi(2) (*n* = 7, ***C***). ***D***, ***E***, Flies incubated 25 min with IBMX (200 μM) followed (*continued in page 11*). by nicotine application (*n* = 6, ***D***) and IBMX spontaneous Ca^2+^-response (*n* = 4, ***E***). ***F,*** Bioluminescent Ca^2+^-activity with upregulated degradation of cAMP through an overexpression of *dnc*-PDE using *UAS-dnc* transgenic construct (*n* = 8). ***G***, ***H***, Bioluminescent nicotine-evoked Ca^2+^-activity with downregulated cAMP production, combined with downregulated cAMP degradation in *rut^2080^*;*dnc*-RNAi(1) (*n* = 7, ***G***), and in *rut*-RNAi(2);*dnc*-RNAi(2) (*n* = 7, ***H***). ***I,*** Bioluminescent image (accumulation time: 120 s) of the nicotinic Ca^2+^-response of a typical fly for each genotype. ***J***, ***K***, Total number of photons during the nicotine response in the CCB (***J***) and in the ML (***K***). ***L***, ***M***, Total duration of the response in the CCB (***L***) and in the medial lobe (***M***). Values are mean ± SEM. Statistics: same as for Figure 2.

Similarly, the spatial resolution obtained at the level of the MB lobes did not allow us to precisely discriminate different subneuronal populations from each other. Therefore, the α/α’ lobes are considered altogether as the vertical lobe (VL), while the β/β’ and γ lobes are considered altogether as the medial lobes (ML) in this study. Moreover, due to the position of the fly’s head and the recording angle, the VLs partly overlapped with the peduncles of the MBs. Thus, in order to avoid any bias in subsequent analysis, we only quantified the response in the CCB and ML. In summary, the first component of the response corresponds to the calyx (dendritic branches), whereas the second component corresponds to the cell bodies (Fig. 1*E*). The response in the ML (Fig. 1*E*, red curve) was delayed compared to the CCB response, and was composed of a single peak of approximately 1100 ph/s, which occurred roughly 10 s after response initiation. The ML response lasted for about 55 s in total. We also quantified the total number of emitted photons for the response in the CCB and ML. The TP average was ∼39000 photons from the CCB and ∼13000 from the ML (see Fig. 3). Finally, to confirm that this robust Ca^2+^-response does not significantly vary with genetic background, results were obtained with additional control lines (VDRC-GD-60000 and VDRC-KK-61000), which were recorded and then shown to share the same characteristics as the CS trans-heterozygotes flies (Fig. 2*I−J*,*L−O*, blue bars).

**Table 1:** Statistical significance of all different tested conditions (histograms of Figs. 2, 3, 4, 5, 7)

		Total Photon		Duration	
Comparison back to control	*N*	CCB	Medial	CCB	Medial
Control-CS	15	NA	NA	NA	NA
Control-RNAi(1)	17	NA	NA	NA	NA
Control-RNAi(2)	15	NA	NA	NA	NA
Control ethanol	11	NA	NA	NA	NA
*rut^1^*	6	0.001	0.002	0.020	0.085
*rut^2080^*	7	0.025	0.097	0.225	0.490
*rut-RNAi(1)*	7	0.025	0.268	0.391	0.209
*rut-RNAi(2)*	6	0.017	0.005	0.524	0.047
*UAS-rut*	8	0.023	0.745	0.524	0.254
UAS-dGαs*	7	0.015	0.025	0.029	0.006
Forskolin	6	0.015	0.025	0.225	0.008
8Br-cAMP	7	0.025	0.010	0.481	0.146
*dnc^1^*	14	0.002	0.002	0.974	0.316
*dnc-RNAi(1)*	7	0.005	0.029	0.065	0.010
*dnc-RNAi(2)*	9	< 0.001	0.001	0.065	0.008
IBMX	6	< 0.001	< 0.001	0.002	< 0.001
*UAS-dnc*	8	0.001	0.006	0.351	0.065
*rut2080;dnc-RNAi(1)*	7	0.333	0.242	0.091	0.316
*rut-RNAi(3);dnc-RNAi(2)*	7	0.702	0.025	0.439	0.739
UAS-mC*	10	< 0.001	< 0.001	< 0.001	< 0.001
UAS-R*	8	0.003	0.025	0.017	0.931
UAS-dGαs*;UAS-R*	9	0.778	0.242	0.078	0.056
*CaM-RNAi(1)*	8	0.010	0.404	0.066	0.535
*CaM-RNAi(2)*	8	0.039	0.322	0.481	0.264
*CaM-RNAi(2)* Forskolin	8	0.288	0.495	0.007	0.254
*CaM-RNAi(2);dnc-RNAi(1)*	8	0.014	< 0.001	0.013	0.004
*CaM-RNAi(2)* IBMX	8	0.039	< 0.001	0.346	0.009
*cngc-RNAi(1)*	11	0.001	0.002	0.481	0.870
*cngc-RNAi(2)*	9	0.015	0.007	0.499	0.004
cngc-RNAi(1);UAS-dGαs*	5	0.050	0.527	0.029	0.458
*cngc-RNAi(1)* Foskolin	7	0.911	0.045	0.524	0.725
*cngc-RNAi(2)* 8Br-cAMP	7	< 0.001	< 0.001	0.007	0.008
cngc-RNAi(1);UAS-mC*	8	< 0.001	< 0.001	< 0.001	< 0.001
cngc-RNAi(1);UAS-R*	9	0.025	0.007	0.091	0.338
*cngl-RNAi(1)*	6	< 0.001	0.003	0.002	0.008
*cngl-RNAi(2)*	7	< 0.001	0.001	0.346	0.243
cngl-RNAi(2);UAS-dGαs*	11	0.138	0.072	0.070	0.874
*cngl-RNAi(1)* Foskolin	8	0.444	0.299	0.065	0.163
*cngl-RNAi(1)* 8Br-cAMP	8	0.130	0.033	< 0.001	0.088
cngl-RNAi(2);UAS-mC*	9	< 0.001	< 0.001	< 0.001	< 0.001
cngl-RNAi(2);UAS-R*	9	< 0.001	< 0.001	0.016	< 0.001

**Table 2:** Statistical significance of all different tested conditions (histograms of Figs. 2, 3, 4, 5, 7)

		Total Photon		Duration	
Comparison between		CCB	Medial	CCB	Medial
cngc-RNAi(1);UAS-dGαs*	*cngc-RNAi(1)*	0.966	0.215	0.138	0.108
*cngc-RNAi(1)* Foskoline	*cngc-RNAi(1)*	0.041	0.736	0.296	0.003
*cngc-RNAi(2)* 8Br-cAMP	*cngc-RNAi(2)*	0.256	0.393	0.058	0.942
cngc-RNAi(1);UAS-mC*	*cngc-RNAi(1)*	< 0.001	< 0.001	< 0.001	< 0.001
cngc-RNAi(1);UAS-R*	*cngc-RNAi(1)*	0.898	0.992	0.389	0.060
cngc-RNAi(1);UAS-dGαs*	UAS-Gαs*	< 0.001	0.030	< 0.001	0.004
*cngc-RNAi(1)* Foskolin	Forskolin	0.048	< 0.001	0.571	0.043
*cngc-RNAi(2)* 8Br-cAMP	8Br-AMPc	< 0.001	< 0.001	0.004	< 0.001
cngc-RNAi(1);UAS-mC*	UAS-mC*	0.999	0.649	0.842	0.449
cngc-RNAi(1);UAS-R*	UAS-R*	0.565	0.780	0.493	0.477
cngl-RNAi(2);UAS-dGαs*	*cngl-RNAi(2)*	< 0.001	0.191	< 0.001	0.005
*cngl-RNAi(1)* Foskolin	*cngl-RNAi(1)*	< 0.001	0.093	< 0.001	< 0.001
*cngl-RNAi(1)* 8Br-cAMP	*cngl-RNAi(1)*	< 0.001	0.394	< 0.001	< 0.001
cngl-RNAi(2);UAS-mC*	*cngl-RNAi(2)*	< 0.001	< 0.001	< 0.001	< 0.001
cngl-RNAi(2);UAS-R*	*cngl-RNAi(2)*	0.875	0.287	0.357	0.600
cngl-RNAi(2);UAS-dGαs*	UAS-Gαs*	0.317	< 0.001	0.556	0.009
*cngl-RNAi(1)* Foskolin	Forskolin	0.134	0.005	0.748	0.256
*cngl-RNAi(1)* 8Br-cAMP	8Br-AMPc	0.568	< 0.001	0.015	0.909
cngl-RNAi(2);UAS-mC*	UAS-mC*	0.751	0.945	0.971	0.146
cngl-RNAi(2);UAS-R*	UAS-R*	0.395	0.033	0.957	0.002
UAS-Gas*;UAS-R*	UAS-Gαs*	0.057	0.359	0.597	0.396
UAS-Gas*;UAS-R*	UAS-R*	0.004	0.003	< 0.001	0.080

### Decreasing cAMP decreases the Ca^2+^-response

In L&M, the role played by the cAMP pathway within the MB has been extensively studied using genetic and behavioral approaches. However, the involvement of this pathway in modulation of the MB Ca^2+^-response is still only partly documented. Thus, in order to determine the cAMP pathway’s role in the MB Ca^2+^-response modulation, we disrupted it using two different complementary strategies: mutations and targeted MB-specific RNAi (simultaneously under the control of the same P[Gal4] GFP-aequorin driver line: OK107). We first tested two different mutants of the *rut* gene encoding AC: the loss of function *rut^1^*(Feany, 1990) and *rut^2080^*, a P-element insertion (Levin et al., 1992). *rut^1^* showed a global decrease in activity (Fig. 2*A*), despite a similar pattern of activity to control flies, with two components in the CCB and one in the ML. The first component had a mean value of ∼880 ph/s, while the second had a mean of ∼600 ph/s. These values correspond to ∼40% of the average control response intensity. The response amplitude in the ML was about half (51%) as strong as that of control flies. The total duration was slightly diminished in the CCB (59% of the control response), but not significantly affected in the ML (Fig. 2*L−M*). The TP was the most affected parameter, with decreases of 32% in the CCB and 36% in the ML, compared to control *rut^1^* flies (Fig. 2*L, M*). The second mutant *rut^2080^* (Fig. 2*B*) had a 61% decreased response amplitude in the CCB compared to the control, while the response amplitude in the ML was not significantly reduced. The TP was significantly reduced only in the CCB (Fig. 2*L,M*). Finally, response duration was not significantly reduced in *rut^2080^*(Fig. 2*N*,*O*).

The cAMP pathway is a ubiquitous signaling pathway involved in several other critical processes, such as apoptosis (Zhang et al., 2006) and cellular fate (Bilodeau et al., 2000). Hence, in order to overcome unspecific effects of the pathway, potentially induced by expression outside the desired structure, we disrupted it locally only in the MBs using targeted RNAi, under the control of the Gal4/UAS system (Brand and Perrimon, 1993). In addition, since each RNAi could have higher or lower efficiency, we used two independent RNAi constructs. In general, the two RNAis gave similar results (Fig. 2*C*,*D*). In CCB, they reduced the nicotine-evoked response by ∼73% compared to controls, without disturbing either the kinetic properties or the total response duration. Surprisingly, in ML, the first RNAi (RNAi-1) did not change the Ca^2+^-response, whereas with the second RNAi (RNAi-2), the response was reduced to ∼44% compared to the control. Altogether, these data show that a defect in *rut-*AC activity, resulting in decreased cAMP, leads to an overall Ca^2+^-response decrease in the MBs, without modifying its general kinetic proprieties.

### Increasing cAMP increases the Ca^2+^-response

Next, we looked at the inverse effect: an increase of cAMP production. First, we overexpressed the cAMP-producing enzyme *rutabaga* using the Gal4-UAS system (Brand and Perrimon, 1993). *UAS-rut* has been commonly used in order to rescue *rut* mutations (Zars et al., 2000). With this approach, the flies show a significant increase of 152% of the CCB response both in amplitude and the TP, while the other parameters remained unmodified (Fig. 2*E*,*L−O*). We then targeted UAS-G_αs_* expression to the MBs. Gα_s_* is a mutated, constitutively active form of Gα_s_ protein, which results in *rut* upregulation. Gα_s_* has been successfully used to disrupt olfactory L&M in MBs (Connolly et al., 1996). Flies expressing the UAS-Gα_s_* showed a significant increase in the Ca^2+^-response (Fig. 2*F*). The first component in the CCB and the response in the ML showed an increase of 162% and 156%, respectively, compared to control amplitude. The second component was less affected in the CCB, showing ∼125% increase. Both TP and duration were significantly increased in the CCB and ML (Fig. 2*L−O*).

AC stimulation in flies expressing *UAS-rut* and UAS-Gα_s_* is constitutive (chronic) and independent of any physiological regulation. To assess the effect of acute AC stimulation, we pharmacologically induced cAMP production using forskolin, an AC stimulator (de Souza et al., 1983). Forskolin (13 μM) dissolved in ethanol was applied 10 min prior nicotine application. The results obtained by application of the vehicle alone (ethanol) on the control lines present the same characteristics than the normal CS trans-heterozygotes flies and showed no spontaneous activity (Fig. 2*J*,*L−O*). Among the eight flies recorded under these conditions, two directly responded to forskolin application. These responses were synchronous in all MB parts, while the CCB response was made up of a single component (data not shown). The flies responding directly to forskolin, prior to nicotine application, were not taken into account for subsequent quantification. The remaining flies that were not responsive to forskolin application had double the amplitude and TP compared to controls, both in CCB and ML (Fig. 2*G*), following nicotine application after 10 min. However, although the response duration increased in the CCB (120%) was not significant, it was significantly increased in the ML (236%) (Fig. 2*N*,*O*). In addition, we stimulated cAMP effectors with the membrane-permeable PDE-resistant cAMP analog 8Br-cAMP (Fig. 2*H*) (Delgado et al., 1991). Flies were incubated in 200 μM 8Br-cAMP for 10 min prior to nicotine application. The results resemble those observed with forskolin, but the response kinetics was different. The peak was delayed in both the CCB and the ML, suggesting that response kinetics might be particularly sensitive to cAMP degradation.

Previous experiments resulted in chronic or acute disruption of cAMP synthesis regulation. Conversely, in order to increase the quantity of cAMP without impairing *rut* AC, we used a different set of strategies to decrease its degradation through PDE activity. *dnc* was the first L&M mutant described (Byers et al., 1981). The *dnc* gene encodes the only known PDE catalyzing the degradation of cAMP into 5’AMP in the MBs. In order to impair *dnc* in the MBs, we used three different strategies. First, we used the historical hypomorphic mutation *dnc^1^*. Surprisingly, in this mutant, we observed a significant decrease of amplitude and TP (in CCB: amplitude 35%, TP 42%; while in ML: 39% amplitude, TP 45%, compared to control levels) (Fig. 3*A*). The kinetics of the response was similar to controls. Similar to *rut, dnc* is involved in many cellular mechanisms in the adult as well as throughout development (Balling et al., 1987). Next, we looked at the local effect of PDE disruption using RNAi against *dnc*, targeted to the MBs. In these MB-*dnc-*deficient flies, we observed increased Ca^2+^-response in all parts of the MBs (Fig. 3*B*), which is in agreement with the previous experiments of cAMP up-regulation (Gα_s_*, forskolin). Importantly, in the CCB, the amplitude of the two components was significantly increased by 244%, while the amplitude increase was even greater in the ML (300% increase). TP increased by 240% in the CCB and 282% in the ML (Fig. 3*J−M*). Duration was slightly increased (129%) in the CCB, but more affected in the ML (196% increased).

Since the above RNAi results contradicted those obtained from *dnc^1^*, we used two additional independent approaches. First, we undertook a second RNAi (RNAi-2) experiment using a different construct (from NIG Japan). This experiment resulted in a very similar Ca^2+^-response pattern (Fig. 3*C*) to that observed using the first RNAi (Fig. 3*B*) construct. Both components of the CCB were increased by 172%, while the ML increased by 182%. TP was increased by 214% in the CCB and 192% in the ML (Fig. 3*J−M*). The modification of the duration was strikingly similar to the effect observed using the first RNAi. Secondly, we used a pharmacological agent, IBMX (Beavo et al., 1970; Gervasi et al., 2010), to block PDE activity. Flies were preincubated with IBMX (200 μM) for 10 min prior to nicotine application. Similar to forskolin, we observed spontaneous activity in four of 10 flies. These responses had slight variability in amplitude and TP, but were similar in kinetics (Fig. 3*D*, Movie 1). In the four flies showing IBMX-induced activity, Ca^2+^-response pattern was rather different from the nicotine-evoked response profile. It consisted of one fast-synchronous component in the ML and CCB without clear propagation or rebound. The six remaining flies that were nonresponsive to IBMX showed a significantly increased nicotine response (Fig. 3*E*). This increase was again very comparable to the effect of the RNAi. In the CCB and the ML, the amplitude was increased by 248%. TP increased by 240% in the CCB and 275% in the ML (Fig. 3*J−M*). Durations were not significantly affected in the CCB and the ML. Except for *dnc^1^*, all results show a direct link between Ca^2+^-response modulation and cAMP levels for both acute and chronic upregulation. Although this increase appears to be a general phenotype, careful observation shows that acute and chronic modification of cAMP levels leads to slightly different effects. Indeed, acute activity, using pharmacological agents (IBMX, forskolin), did not increase response duration in the CCB, whereas chronic activity, using genetic approaches such as G_αs_* and *dnc*-RNAi, increased this duration. These results suggest the existence of different molecular mechanisms in Ca^2+^ modulation in acute versus long-term cAMP increase. In order to further investigate the effect of a *dnc* disturbance, we overexpressed the gene using the *UAS-dnc* construct (cDNA), which is commonly used to rescue *dnc* mutations (Cheung et al., 1999). In these flies, specifically in the MBs, we observed a radically reduced response (Fig. 3*F*) (even lower than observed with *rut* mutation) with a TP, which reduces to 15% of the control response in the CCB and 16% in the ML (Fig. 3*J−M*). However, this result seems to be in agreement with the results obtained with the *rut*, but in this case (*UAS-dnc*), the duration was also severely reduced. This result is similar to the two independent RNAis and the pharmacology, while completely opposite to what was observed in *dnc^1^* mutation.

Movie 1 *In vivo* bioluminescence imaging of Ca^2+^-responses in the KCs of the MBs induced by nicotine application. On the left, we observe a wild-type control-Canton-S fly; the right corresponds to the so-called spontaneous activity induced by the IBMX application. Each frame represents 1 s of light accumulation and is shifted by 250 ms, seen at 25 frames/s. The light emission is coded in pseudocolors (2-6 photons/pixel) (MP4 = 2.89 Mb). 10.1523/ENEURO.0054-14.2015.video.1

Finally, we generated two different *rut* and *dnc* double-deficient lines by combining the mutation *rut^2080^* with *dnc*-RNAi(1), and *rut*-RNAi(1) with *dnc*-RNAi(2) (Fig. 3*G*,*H*). In both lines, TP and the duration in the CCB were restored (Fig. 3*J*,*K*), while the amplitude was partly restored (75% and 62%, respectively, compared to control). The kinetics of the responses remained disrupted (Fig. 3*G*,*H*). The response in the ML was delayed in both cases, but was differently disrupted in the two double mutant lines. In *rut^2080^*;*dnc*-RNAi, response amplitude was restored to control levels, and in *dnc-*RNAi;*rut-*RNAi, the amplitude decreased by 56%, while TP reduced by 55%, compared to control. Although their kinetics are quite complex, these results suggest that another AC could potentially play a role in Ca^2+^-response modulation, while the Ca^2+^-sensitive *rut* AC is required to obtain a normal response pattern.

### Calmodulin affects the Ca^2+^-response in the CCB, but not in the lobes

**Figure 4:**
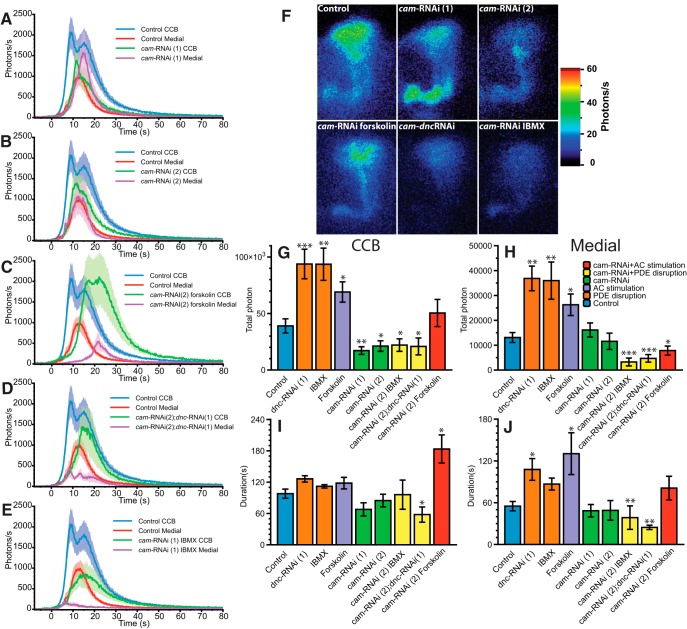
Modulation of the nicotine-evoked Ca^2+^-transient with CaM disturbance. ***A***, ***B***, Bioluminescent Ca^2+^-activity profile evoked by nicotine with a downregulated CaM expression in cam-RNAi(1) (*n* = 8) and cam-RNAi(2) (*n* = 8) flies. ***C,*** Bioluminescent Ca^2+^-activity profile evoked by nicotine with a downregulated CaM expression in cam-RNAi(2) and stimulated AC flies incubated 10 min with forskolin (13 μM) (*n* = 8). ***D***, ***E***, Bioluminescent Ca^2+^-activity profile evoked by nicotine with a downregulated CaM expression in cam-RNAi(2) and a blockade of the PDE with *dnc*-RNAi(1) flies (*n* = 8, ***D***) or flies incubated 10 min with IBMX (200 μM, *n* = 8, ***E***). ***F,*** Bioluminescence image (accumulation time: 120 s) of the nicotinic response in a representative fly for each genotype. ***G***, ***H***, Total number of photon during the nicotine response in the CCB (***G***) and in the ML (***H***). ***I***, ***J***, Total duration of the response in the CCB (***I***) and in the medial lobe (***J***). Values are means ± SEM. Statistics: same as for Figure 2.

cAMP is at the center of the theoretical model of the coincidence detector. This model places MBs at the intersection of two kinds of stimuli: the unconditioned stimuli (negative or positive reinforcement) and the conditioned stimulus (generally the odors) (Heisenberg, 2003; Davis, 2005). Theoretically, this integration relies on the dual regulation of *rut* AC by Ca^2+^-calmodulin (CaM) (supposedly in association with the nAchR odor response) present in the CCB, and the G-protein (G_s_) coupled to a metabotropic receptor (associated with neuromodulators released by reward or nociceptive pathways) present in the MB lobes (Heisenberg, 2003; McGuire et al., 2005; Keene and Waddell, 2007; Waddell, 2010). Despite a number of studies supporting this model, the importance of CaM on Ca^2+^-response modulation has not been directly demonstrated *in vivo* so far, although a *rut* CaM-independent deficiency is associated with defective memory (Livingstone et al., 1984). Hence, to explore the role of CaM, we silenced its expression using two independent RNAi constructs. The two RNAi constructs have a rather similar effect on the Ca^2+^-response (Fig. 4*A*,*B*). The response in CCB was delayed and lowered to 75% of control by both RNAi constructs. The response in the ML seemed to be slightly increased with the first RNAi construct, but remained unaffected with the second. TP was decreased (by 50%) in the CCB, but unchanged in the ML (Fig. 4*G*,*H*). The duration did not vary significantly in any part of the MBs with either construct (Fig. 4*I*,*J*). These results indicate that CaM has an effect in the CCB, but not in the MB lobes, and thus suggests a regionalized effect of CaM on the Ca^2+^-response.

Beyond its effect on *rut* AC, CaM is known to interact with several Ca^2+^-regulated proteins through notably the Ca^2+^/calmodulin-dependent protein kinase II (CaMKII) (Yao and Wu, 2001; Lisman et al., 2002; Trudeau and Zagotta, 2003) and *caki* (renamed recently CASK) (Martin and Ollo, 1996; Hodge et al., 2006; Gillespie and Hodge, 2013; Malik et al., 2013), as well as several other targets, both in *Drosophila* and other model organisms. In order to characterize further the putative link between CaM and cAMP, we used two different complementary approaches. First, we applied forskolin on CaM-deficient flies to stimulate directly cAMP production and circumvent the Ca^2+^/CaM effect on *rut*. In this combination, we observed a restoration of the response in the CCB both in amplitude and TP (Fig. 4*C*). However, the kinetics of the response remains disturbed, being characterized by a longer rising time that led to an overall longer duration in the CCB. In addition, surprisingly, in the ML we observed a striking diminution of the TP to 59% (Fig. 4*H*) and the amplitude to 44% of controls (Fig. 4*C*).

Second, we used *dnc*-RNAi or IBMX in a CaM-deficient context, thus combining the disruption of the degradation of cAMP with a decreased endogenous production of cAMP due to the deficit of Ca^2+^/CaM stimulation on *rut*. In both cases, these combinations failed to restore the control response level in the CCB (Fig. 4*D*,*E*). The response of ML were even weaker than previously observed with forskolin (IBMX+CaM-RNAi = 25%; *dnc*-RNAi+CaM-RNAi = 36%) (Fig. 4*G−J*). However, the outcome effects on the Ca^2+^-response of these three different experimental approaches are different: the first case (CaM-RNAi alone; Fig. 4*A*,*B*) yields a decrease in the CCB without affecting the ML; the second case (CaM-RNAi + forskolin; Fig. 4*C*) yields a restoration of the Ca^2+^-response in the CCB but a striking decrease in the ML; and the third case (CaM-RNAi + IBMX or *dnc*-RNAi; Fig. 4*D*,*E*) yields no restoration of the Ca^2+^-response in the CCB, but rather a striking decrease in the ML. In summary, altogether these three approaches share a differential effect between the CCB and the lobes, confirming that CaM effects are dissociated between these two compartments and therefore might be regionalized in the MB.

### PKA is a major modulator of the Ca^2+^-response

**Figure 5:**
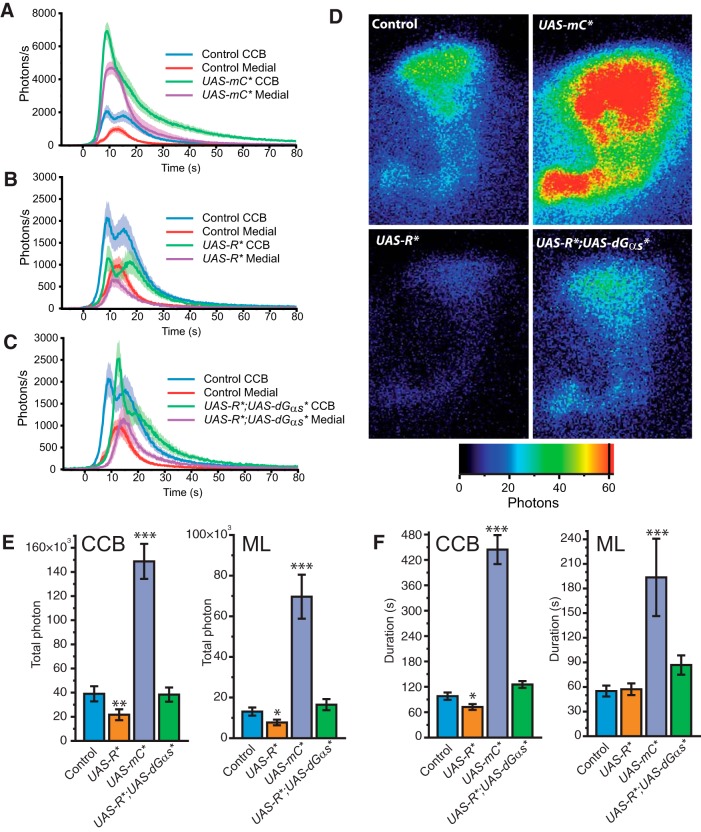
Modulation of the nicotine-evoked Ca^2+^-transient with PKA disruption. ***A−C***, Bioluminescent Ca^2+^-activity evoked by nicotine with a constitutively activated PKA in mC* transgenic flies (*n* = 10, ***A***), with downregulated PKA production in R* transgene flies (*n* = 8, ***B***) and a high level of cAMP with PKA blocked in R* combined with a G_αs_*(*n* = 9, ***C***). ***D***, Bioluminescent image (accumulation time: 120 s) of the nicotinic response in a typical fly of each genotype. ***E***, Total number of photons during the nicotine response in the CCB and in the ML**. *F,*** Total duration of the response in the CCB and in the ML. Values are mean ± SEM. Statistics: same as in Figure 2.

The cAMP-dependent PKA plays several roles in many species, particularly neural plasticity in mammals (Nguyen and Kandel, 1996; Kandel, 2012). The impairment of PKA activity in *Drosophila* has been related with strong L&M phenotypes (Skoulakis et al., 1993; Yamazaki et al., 2010). cAMP was also shown to locally regulate PKA in MBs (Gervasi et al., 2010). PKA is a multimeric holoenzyme composed of two regulatory and two catalytic subunits. Following activation by cAMP, PKA plays various roles such as K^+^-channel phosphorylation (Drain et al., 1994; Esguerra et al., 1994) and transcriptional regulation through cAMP response element-binding protein (CREB) (Yin et al., 1995b). In order to assess PKA’s role in Ca^2+^-response regulation, we first used UAS-mC*, a constitutively active catalytic subunit of murine PKA (Li et al., 1995). This constitutively active subunit was previously shown to impair sleep when specifically expressed in the MBs (Joiner et al., 2006; Pitman et al., 2006). Flies expressing the mC* in the MBs showed a significant increase in the Ca^2+^-response (Fig. 5*A*, Movie 2). The first exponential phase in the CCB culminated with an intensity of 287% compared to controls. Consequently, the second component was almost invisible, since it merged with the decreasing phase of the first exponential. The response in ML was increased by 351%. The responses were also significantly prolonged in the calyx, albeit to a weaker level (100 ph/s), with an average duration of 444 s in the CCB (450% increase) and 193 s in the ML (350% increase), following response initiation (Fig. 5*F*). Conversely, TP had a significantly greater increase (532%) in the ML compared to the CCB (381% increase) (Fig. 5*E*). Next, to assess if blocking PKA yields an opposite effect on Ca^2+^-response, we expressed a mutated regulatory subunit of PKA, UAS-R*, which constitutively blocks the catalytic subunit by competing with the endogenous regulatory subunit (Li et al., 1995). These flies not only had reduced Ca^2+^-responses (Fig. 5*B*), but these responses were delayed and their intensity halved for both components in the CCB. In contrast, the ML responses did not appear to be delayed, although their intensity decreased. TP also halved in CCB and ML, but its duration was diminished only in the CCB (Fig. 5*E*,*F*).

Movie 2 *In vivo* bioluminescence imaging of Ca^2+^-responses in the KCs induced by nicotine application on a fly expressing the UAS-mC*. Left, Control-CS (same fly as in Movie 1). Right, A fly expressing the UAS-mC*, a constitutively activated PKA. We remark that the level and the duration of activity is importantly increased in mC* fly. However, for the sake of the visualization of the movie, notice that the duration of this movie does not exactly correspond to the calculated duration reported in Figure 5*F*, because the accumulation time of the signal is settled and displayed differently. Each frame represents 1 s of light accumulation and is shifted by 250 ms, seen at 25 frames/s. The light emission is coded in pseudocolors (2-6 photons/pixel) (MP4 = 2.89 Mb). 10.1523/ENEURO.0054-14.2015.video.2

Given similar results for AC and PKA regulation, we next studied the effect of blocking PKA under high cAMP levels, the rationale being that this would potentially reveal a direct putative role of cAMP on Ca^2+^-response, independent of its effect via PKA. In order to do this, we coexpressed G_αs_* and R* in KCs. Interestingly, the double-transgenic flies displayed an intermediate phenotype (Fig. 5*C*). The response was delayed in the CCB, similar to the R* flies, but its intensity reached a level roughly similar to controls in both CCB and ML. The results obtained with PKA impairment reveal a strong positive influence of this effector on Ca^2+^-response globally. However, the results with the double-transgenic flies led us to question whether an additional cAMP-regulated PKA-independent mechanism may regulate the Ca^2+^-response.

### The Ca^2+^-response is modulated through CNGs

**Figure 6:**
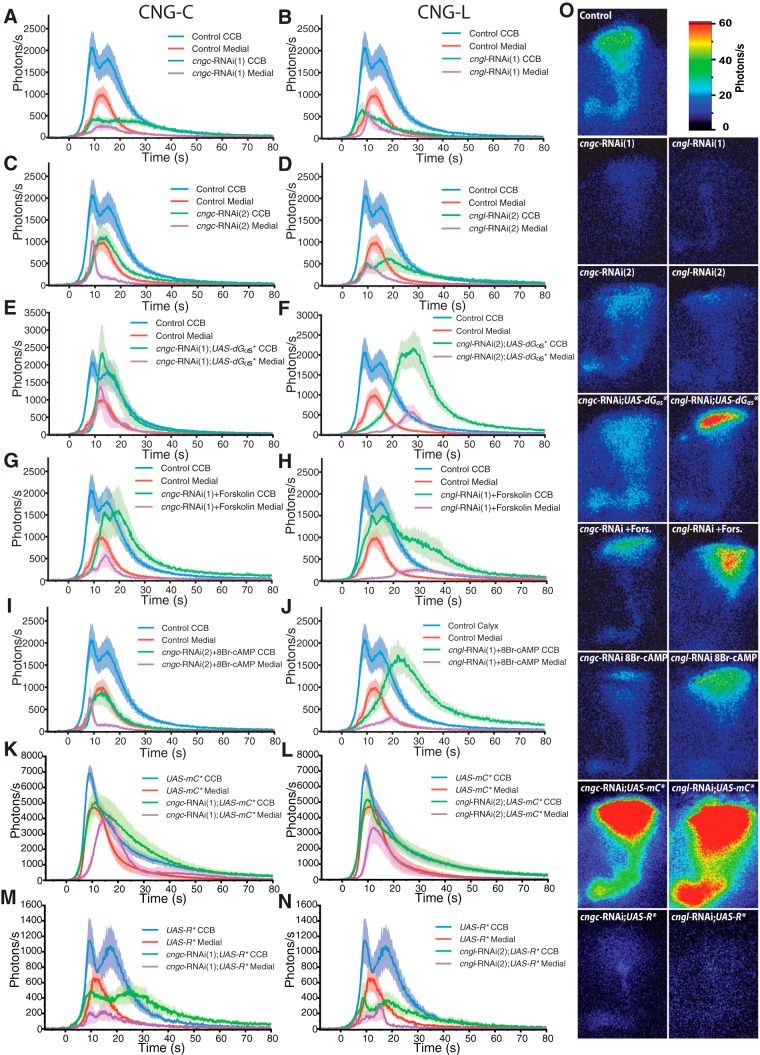
Modulation of nicotine-evoked Ca^2+^-transient with CNG disruption. ***A−D***, Bioluminescent Ca^2+^-activity evoked by nicotine in CNGs knocked-down using the following: *cngc*-RNAi(1) (*n* = 11, ***A***), *cngc*-RNAi(2) (*n* = 9, ***B***), *cngl*-RNAi(1) (*continued in page 16*). (*n* = 6, ***C***), and *cngl*-RNAi(2) (*n* = 7, ***D***). ***E***, ***G***, ***I***, Bioluminescent Ca^2+^-activity in CNGC knockdown with upregulated cAMP production in G_αs_* (*n* = 5) transgenic flies (***E***), flies incubated for 10 min with forskolin (13 μM, *n* = 7, ***F***), and flies incubated for 10 min with 8Br-cAMP (200 μM, *n* = 7, ***I***). ***F***, ***H***, ***J***, Bioluminescent Ca^2+^-activity in CNGL knockdown with upregulated cAMP production in G_αs_* (*n* = 11) transgenic flies (***F***), flies incubated for 10 min with forskolin (13 μM, *n* = 8, ***H***), and flies incubated for 10 min with 8-Br-cAMP (200 μM, *n* = 8, ***J***). ***K***, ***M***, Bioluminescent Ca^2+^-activity in CNGC knockdown with upregulated PKA activity in UAS-mC* (*n* = 8, ***K***) and downregulated PKA activity in UAS-R* (*n* = 9, ***M***). ***L***, ***N***, Bioluminescent Ca^2+^-activity in CNGL knockdown with upregulated PKA activity in UAS-mC* (*n* = 9, ***L***) and downregulated PKA activity in UAS-R* (*n* = 9, ***N***). ***O***, Bioluminescent image (accumulation time: 120 s) of the nicotine-evoked response in a typical fly of each genotype.

In order to explore the cAMP-dependent, PKA-independent effect in MB response modulation, we looked for direct potential targets of cAMP. Amongst them, the CNGs are a class of channels that can be opened by cAMP or cGMP (Baumann et al., 1994). These channels are mainly permeable to Ca^2+^, although they are also permeable to most other cations. Four genes in *Drosophila* are predicted to encode CNGs: *cngc, cng-b, cngl*, and *cg42260* (Kaupp and Seifert, 2002). For our experiments, we used RNAi against two different CNGs expressed in the adult brain: *cngc* and *cngl* (Baumann et al., 1994; Miyazu et al., 2000). CNGC has mostly been studied for its role in the response to hypoxia (Vermehren-Schmaedick et al., 2010). It is expressed in the adult brain, and was shown to be very responsive to cGMP, and to a lesser extent to cAMP (tenfold less). Calcium flux, through this channel, is also blocked in a voltage-dependent manner (Baumann et al., 1994). We used two different RNAi constructs against *cngc*, which led to concordant results for TP and duration, but with different kinetic phenotypes (Fig. 6*A*,*C*). Both RNAi showed a decreased TP in the CCB (38% and 47%) and the ML (41% and 45%), but duration was only affected in the ML (45%) of *cngc*-RNAi(2). In *cngc*-RNAi(1), the TP was decreased, but the overall response followed similar kinetics as controls (two components in the CCB and a delayed response in the ML) (Movie 3). *cngc*-RNAi(2) had a disrupted waveform: the ML response peaked before the CCB and was followed by a low-activity tail. In addition, it was narrower than the control response. The CCB response in the second RNAi was only made of one component, which had a longer rising time. These different effects between the two RNAi constructs may potentially be due to different expression levels.

Movie 3 *In vivo* bioluminescence imaging of Ca^2+^-responses in the KCs induced by nicotine application on a fly expressing the cngc-RNAi(1). Left, Control-CS fly. Right, A fly expressing the cngc-RNAi(1). We remark that the level and the duration of activity are importantly reduced in this fly. Each frame represents 1 s of light accumulation and is shifted by 250 ms, seen at 25 frames/s. The light emission is coded in pseudocolors (2-6 photons/pixel) (MP4 = 2.89 Mb). 10.1523/ENEURO.0054-14.2015.video.3

In order to directly determine the role of cAMP on CNGC, we increased cAMP in a *cngc* knocked-down background using three independent methods. First, we coexpressed Gαs* with RNAi(1) (Fig. 6*E*), which led to increased amplitude, similar to control levels, but not to Gαs* levels (compared to Fig. 2*F*). Furthermore, values for TP and duration were in between control flies and those that had *cngc*-RNAi alone. We then applied the pharmacological agents forskolin and 8Br-cAMP to the *cngc*-RNAi flies. Forskolin (Fig. 6*G*) led to a very similar phenotype as *cngc*-RNAi(1);Gαs* flies, except for the response in the ML, which was decreased compared to controls, but similar to the *cngc*-RNAi(1) response. Next, we applied 8Br-cAMP to *cngc*-RNAi(2) (Fig. 6*I*) flies, which resulted in a response that shared all the characteristics of the second RNAi. In summary, the combination of increased cAMP and *cngc*-RNAi leads to an effect that is in between controls and *cngc*-RNAi on its own. In other words, it never restores the enhanced cAMP phenotype to the CCB or ML, suggesting that the CNGC channel plays an early and crucial role in the Ca^2+^-response modulated by cAMP.

**Figure 7:**
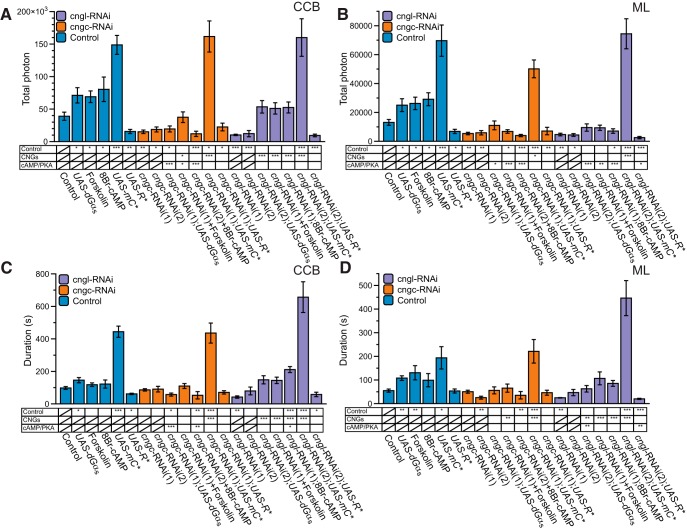
Quantification of the modulation of the nicotine-evoked Ca^2+^-transient with CNGs disruption ***A***, ***B***, Total number of photons during the nicotine response in the CCB (***A***) and in the ML (***B***). ***C***, ***D***, Total duration of the response in the CCB (***C***) and in the ML (***D***). Values are mean ± SEM. Statistics: The tables located under the histograms represent the comparison between different conditions (one modification vs two modifications). The first line is the comparison with the control group. The second line (labeled CNGs) compares groups exposed to two modifications (RNAi + transgenes or RNAi + pharmacology) with results obtained for its single-channel RNAi counterpart (either *cngc-*RNAi or *cngl-*RNAi). The third line (labeled cAMP/PKA) compares the groups exposed to two modifications with their respective single modification affecting either cAMP or the PKA. Statistical tests are the same as for Figure 2.

Finally, we investigated the physiological effects of the combination of the depletion of CNGC and the PKA transgenic construct: UAS-mC* and UAS-R*. With the coexpression of UAS-mC* (the constitutively activated PKA subunit) and *cngc-*RNAi(1), the nicotinic responses are very close to those of the mC* flies, both in duration and TP. However, the double transgenes (*cngc-*RNAi(1);UAS-mC*) show some slight modification of their kinetics (Fig. 6*K*), which are globally slower, and also exhibit a significant diminution of the TP in the medial lobe (Fig. 7*B*). We then suppressed the PKA activity using the combination of UAS-R* and *cngc-*RNAi. While double-transgenic flies show a kinetic phenotype very close to what was observed with *cngc-*RNAi(1) alone (Fig. 6, *A* vs *M*), their TP and response duration remained at the same value as observed in both single-transgenic flies (which were themselves very similar to each other) (Fig. 7). The results obtained with the manipulation of PKA suggest that *cngc* is not necessary for the amplification of the response observed in UAS-mC* flies while it remains critical for the kinetic properties. UAS-R* experiments seem to indicate that we already obtained the maximal effect (the minimal Ca^2+^-response) using *cngc* knockdown.

CNG-like (CNGL), encoded by *cngl*, is another cAMP/cGMP-sensitive channel that is expressed at high levels in the central brain of *Drosophila* (Miyazu et al., 2000). It shares most structural characteristics of CNGC, except for the aspartic acid residue found in other CNG channels that is necessary for cGMP selectivity (Varnum et al., 1995). This residue is substituted by a valine in CNGL channels (Miyazu et al., 2000). No data on CNGL physiology or ion selectivity are available yet, but the structural features suggest that, similar to CNGC, CNGL contains a voltage-sensitive region that is permeable to most cations, and probably mainly to Ca^2+^ (Miyazu et al., 2000). We used two independent RNAi constructs directed against *cngl* that gave roughly similar results (Fig. 6*B*,*D*). The amplitude and TP in both RNAi were dramatically reduced in the CCB (26% and 31%, respectively) and the ML (35% and 33%, respectively). The kinetic properties were slightly different between the two RNAi lines: they both followed a two-component response, but the rising phase and overall duration were longer (a duration comparable to control) in RNAi(2). Next, we combined CNGL impairment with cAMP stimulation using the same tools as was previously done with CNGC (Gαs*;*cngl*-RNAi(2) (Fig. 6*F*). Similarly, treating *cngl*-RNAi(2) flies with either forskolin or 8Br-cAMP had a strikingly similar effect on the responses (Fig. 6, *H* and *J* vs *F*). The CCB amplitude was restored, but the rising phase was longer than controls, increasing the duration of the response. The response in the ML was significantly decreased and delayed compared to controls. TP in the CCB was between 398% and 509% higher than in flies containing *cngl-*RNAi alone (Fig. 7), and in between TP values of the control and cAMP-enhanced flies. In contrast, the response in the ML ranged from 24% to 38% of that of the cAMP-enhanced flies and was between *cngl-*RNAi and control levels. The results with CNGL knocked-down suggest that this channel has a major role in response generation (result with RNAi alone), but can be supplemented in the CCB by enhancement of another class of channels sensitive to cAMP. However, CNGL seems to be essential for propagating the response to the ML. A noticeable feature of both CNGs is that their phenotype is more severe than that observed in the *rut*-deficient files, suggesting that either other ACs are involved in CNG stimulation or they play a similar role as the voltage-gated cation channels through their voltage-sensitive characteristics.

Finally, as previously performed with *cngc*, we combined the *cngl*-RNAi with UAS-mC* and UAS-R*. As observed with *cngc*-RNAi(1);UAS-mC* flies, the response of the *cngl-*RNAi(2);UAS-mC* flies were very similar to UAS-mC* flies, with few modifications of the kinetic property of the response; the response amplitude seemed to be lower than in mC* (Fig. 6*L*) flies, but this diminution is compensated by an increased duration of response tail (Fig. 7*C*,*D*), giving a final TP at the same level as observed in mC* transgenic flies (Fig. 7*A*,*B*). The effect of the combination of UAS-R* and *cngl-*RNAi was also very similar to the phenotype observed in *cngl* knockdown flies both in the CCB and ML (Figs. 6*B*,*N*, 7). These results altogether suggest that the overactivation of the PKA mediate the amplification of the response through a partner, which might play an early role in the response and seems to be independent from *cngl*, which only disturbs the kinetic of the response. As previously seen with *cngc,* the UAS-R* experiments seem to indicate that we already obtained the maximal effect (the minimal Ca^2+^-response) using *cngl* knockdown.

## Discussion

The bioluminescent GFP-aequorin probe (Baubet et al., 2000; Martin et al., 2007) has provided the means to measure functional Ca^2+^-response continuously, over long time periods, with high sensitivity. This probe was previously used to study odor-induced Ca^2+^-response in ORNs (Murmu et al., 2010; 2011) and the MBs, and to detect spontaneous Ca^2+^-activity in neurons and glial cells (Minocci et al., 2013). In this study, we used this approach to simultaneously record and temporally correlate the responses of the CCB and lobes, and vizualized Ca^2+^-activity propagation in the axonal projections within the MB lobes.

As reported in primary pupal cultures of KCs (Jiang et al., 2005; Campusano et al., 2007), our experiments show that bath application of nicotine mimics the endogenous Ach-neurotransmitter-induced Ca^2+^-response, and thus can be used as a faithful and reliable agonist. We show that the nicotine-evoked Ca^2+^-response in the calyx/cell-bodies consists of two successive components: the first corresponds to the response in the calyx, while the second corresponds to the response in the cell bodies. However, multiple factors such as the complex 3D architecture and the angle at which the MBs are visualized lead to a partial overlap of these two components. Moreover, the combination of the design of the approach and the relatively slow perfusion kinetics of nicotine always leads to partial overlap, both spatially and temporally. Conversely, in the MB lobes, only one component is observed. Finally, given that acetylcholine is the primary excitatory neurotransmitter in the brain, the bath application of nicotine also likely activates other neurons across the brain. Therefore, it is not possible to dissociate which components of the response are due to direct action on MB nAChRs from responses due to activation of afferent circuits. Nonetheless, the RNAi knockdown approach that targets only the MB neurons supports our conclusion that the physiological Ca^2+^-effects described here are indeed due to the disturbance of the given targeted pathway or channels within the MBs.

### The level of the Ca^2+^-response is proportional to the level of cAMP

As mentioned before, although the *dnc* and *rut* mutants have been identified for more than 30 years, their *in vivo* physiological effects on the Ca^2+^-response in the KCs of the adult fly still remain largely unknown. We used several independent strategies (genetic and pharmacology) to demonstrate that the nicotine-induced Ca^2+^-response is proportional to the level (higher or lower) of cAMP. The downregulation through the two alleles of *rut*, or via two different *rut*-RNAis, leads to an ∼50% Ca^2+^-response decrease in both the CCB and lobes. Similarly, the overexpression of *dnc* leads to even a stronger phenotype, suggesting the PDE activity in the response modulation is of critical importance. Conversely, the upregulation using two independent *dnc*-RNAis or by targeted overexpression of *rut* and a constitutively active G-protein subunit (G_αs_*) significantly increases the Ca^2+^-response. Unexpectedly, however, the response is decreased with *dnc^1^*, a contrasting result compared to the other strategies used to increase cAMP. These contradictory results are likely due to defects accumulated during fly development. Indeed, it was reported that *dnc*, which is expressed at various developmental stages, has diverse roles in cells and notably affects the survival of KCs throughout development, leading to smaller CCB in adult flies (Balling et al., 1987).

We also use a pharmacological strategy to upregulate the level of cAMP. Again, either increasing its synthesis by directly stimulating adenylyl cyclase using forskolin or diminishing its degradation using IBMX yields similar results: a huge increase of the Ca^2+^-response. Thus, the similar results obtained by these independent approaches demonstrate that cAMP levels determine the level of the Ca^2+^-response. Based on literature describing L&M defects caused by disruption of the cAMP pathway, it seems that fine regulation of the level of cAMP is the crucial parameter. This is because both its decrease or its increase disrupts L&M, as well as other MB functions such as centrophobism (Besson and Martin, 2005; Lebreton and Martin, 2009) or sleep (Joiner et al., 2006). Research to date has only investigated the L&M function of *rut*; however, as already discussed (Tomchik and Davis, 2009; Gervasi et al., 2010), a number of other putative ACs have been reported in the *Drosophila* genome (DAC39E, DAC78C, DAC76E, CG32158, CG32301, CG32305). Thus, it is possible that these additional noncharacterized ACs could be active in the MB lobes or the CCB.

### Acute increase of cAMP triggers a transient Ca^2+^-response in the KCs

In this study, we used two fundamentally different and complementary methods to disrupt cAMP pathway: on one hand, we used the genetic approach (mutants, targeted overexpression, and targeted RNAi), which induce chronic modifications of the pathway; and on the other hand, we used the pharmacological approach, which corresponds to an acute effect (stimulation or blockade). Interestingly, an acute increase of cAMP synthesis, or reducing its degradation by pharmacological approaches (forskolin or IBMX), induced Ca^2+^-activity in the MBs. In contrast, chronic dysregulation (mutant, targeted RNAi) did not induce any spontaneous Ca^2+^-activity. These results suggest that chronic misregulation of cAMP can be (at least partially) compensated by other mechanisms (e.g., signaling pathway partners such as PKA, or upregulation or downregulation of various channels) in neurons, while an acute modification is not compensated, and therefore is sufficient to trigger a response in the KCs. We also found that a chronic upregulation of cAMP in the calyx led to a prolonged response (*dnc*-RNAi, Gα_s_-transgene), possibly through the effect of PKA, while acute upregulation (by pharmacology: IBMX and forskolin) does not affect the duration. This suggests that the effect might be, at least partially, through the CNGs. Interestingly, it was reported that KCs cultured from late stage pupae showed spontaneous Ca^2+^-transients in a cell autonomous fashion (Jiang et al., 2005). In addition, functionally behavioral genetic approaches demonstrated that synaptic transmission between KCs and their downstream partners is important in memory retrieval, but not necessary for memory acquisition or storage (Dubnau et al., 2001). Consequently, we hypothesize that this spontaneous triggering following an acute increase in cAMP, either pharmacologically evoked or naturally occurring, could represent a molecular and cellular mechanism for reminiscence or retrieval, since the KCs seem to be able to activate themselves spontaneously (cell autonomously) without any afferent stimuli.

### PKA-dependent and -independent effect of cAMP

PKA is the best known effector of cAMP. The constitutive activation of the catalytic subunit strikingly increases the Ca^2+^-response both in CCB and lobes. Moreover, it significantly prolongs the response duration by up to 444 s in the CCB (more than fourfold). In contrast, blocking the regulatory subunit using a dominant negative form (UAS-R*) decreases the Ca^2+^-response. However, the latter also delays the response in the CCB. PKA likely acts by phosphorylating the K^+^-channels (Delgado et al., 1992; Brüggemann et al., 1993; Zhou et al., 2002) and/or regulating CREB (Yin et al., 1995a; 1995b). The chronic inactivation of PKA may have phosphorylated some K^+^-channels, resulting in a modified resting membrane potential. This, in turn, may have led to less excitable cells, which consequently delayed the Ca^2+^-response. Additionally, the residual response following PKA blockade (UAS-R*) revealed that cAMP can act by itself, and thus represents its PKA-independent effect. To corroborate these results, we increased the production of cAMP in PKA-blocked flies, which indeed increased the Ca^2+^-response (UAS-R*;UAS-Gα_s_). Furthermore, the knockdown of the different CNGs, which decreased the Ca^2+^-response, suggests that these channels play a crucial role in KC responsiveness. Moreover, cAMP supplementation is never sufficient to restore the kinetic properties of the response in a CNG-deficient context. The different response patterns displayed after cAMP enhancement in a CNG knockdown context suggest that the two different CNGs are playing segregated and sequential roles in cAMP-dependent regulation of MBs responsiveness: CNGC seems to play an early role on the overall response, while CNGL seems to play its role mainly in the medial lobe (for example, response level only partly rescued in the medial lobes). The overactivation of PKA in CNG-deficient context [*cng*-RNAi(1) or *cng*-RNAi(2);UAS-mc*] does not restore the kinetic proprieties of the responses but completely restores the quantitative parameters (TP and duration) observed with an overactivation of the PKA alone, suggesting that CNGs are not required for PKA’s modulation of the response but are critical for its kinetics. Inversely, the blockade of the PKA activity in CNG-deficient flies [*cng*-RNAi(1) or *cng*-RNAi(2);UAS-R*] yields to a CNG phenotype, confirming that the CNG-dependent modulation of Ca^2+^-response is independent of the PKA.

### The differential effect of calmodulin between the CCB and the lobes

The *rut-*AC is a Ca^2+^/calmodulin-dependent enzyme (Livingstone et al., 1984; Levin et al., 1992). However, a function of CaM, *per se,* within the KCs has not yet been directly described. Here, we functionally demonstrated that CaM knockdown results in a segregated and regionalized effect. While it significantly decreases the Ca^2+^-response in the CCB, it does not affect the responses in the lobes. This effect suggests that either its modulation is necessary for Ca^2+^-responsiveness in the CCB, but not in the MB lobes, or a calmodulin-independent AC activation occurs in the lobes, putatively through another AC. Rut protein is present in the α,β,γ lobe branches (Han et al., 1992), while functional subdivisions in cAMP synthesis within the MBs has been reported (Gervasi et al., 2010). Since Rut-AC can be activated either by Ca^2+^/calmodulin or via G-protein stimulation, a first hypothesis could be that in the CCB, Rut-AC activation occurs through calmodulin, while in the lobes it could be activated through G-proteins.

**Figure 8:**
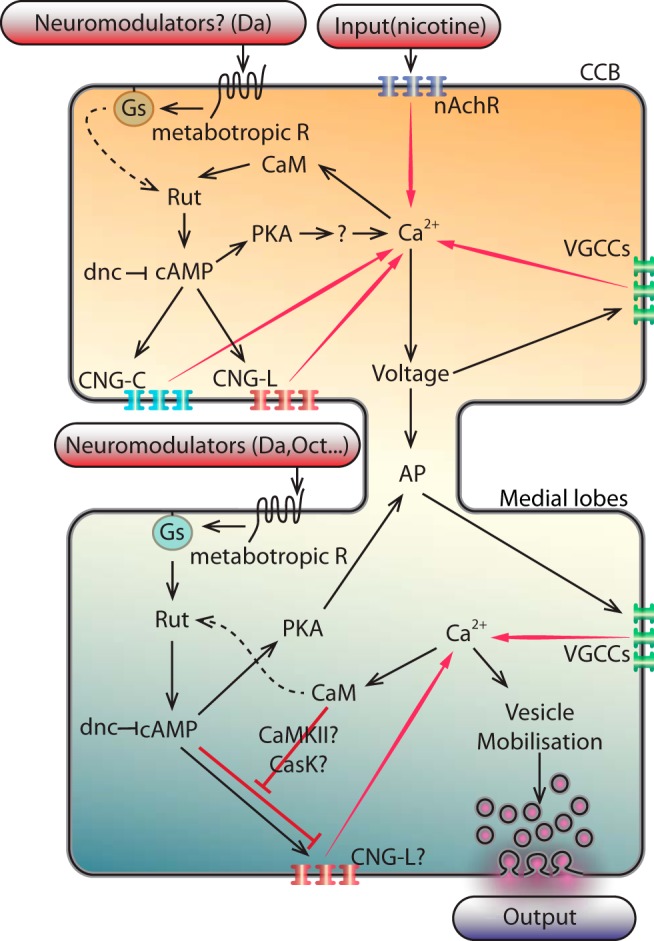
Schematic integrative model of interactions of the different partners involved in the Ca^2+^-response modulation in the CCB and the medial lobe. In the CCB: the conditional stimulus (e.g., an olfactory stimulus) triggers nicotinic inputs, which activate the nAchR located on the KCs of the MBs, allowing the Ca^2+^-entry. Calcium binds to CaM and subsequently activates the production of cAMP by RUT. In agreement with the coincidence detector model, in certain conditions, e.g., when the conditional stimulus is simultaneously applied with an unconditional stimulus (e.g., a nociceptive electric shock), the dopaminergic receptors could costimulate the *rut*-AC to increase further the cAMP level through G_αs_. The resulting increase in cAMP stimulates the PKA as well as the CNGs (CNGC and CNGL), which both participate in amplifying the Ca^2+^-entry. At the same time, the PKA allows the Ca^2+^-entry and/or the persistence of the Ca^2+^-entry, likely by affecting the repolarisation of the cells, possibly through the K^+^-channels. In parallel and simultaneously, the Ca^2+^-entry modifies the voltage of the cells that allows the voltage-gated calcium channels (VGCC) to also participate to the Ca^2+^-entry. Altogether, these activities trigger actions potentials (APs) that propagate to the lobes. In the medial lobes: at the axon terminals, the APs open the VGCC, allowing Ca^2+^-entry. This Ca^2+^ stimulates the CaM. In parallel or simultaneously (as for instance in certain environmental conditions), a neuromodulator (e.g., dopamine, octopamine) activates a metabotropic receptor, which stimulates a G-protein, and then stimulates the RUT to increase the cAMP. Then, the cAMP stimulates the CNGs (and more likely the CNGL; as suggested by our results in Fig. 6*F,H,J*). Moreover, according to our results (knocking-down the CaM and handling the cAMP level; Fig. 4), we hypothesize that the CaM might act as an inhibitor of the cAMP-stimulation of the CNGL (red line). This could be achieved either through the CaMKII or CASK, since both of them have been implicated in learning and memory, or directly by the CaM on the CNGL (since in some organisms, certain CNGs have been reported to be sensitive to CaM; Kaupp and Seifert, 2002). These successive events lead to the fine tuning of the Ca^2+^-level that mobilize the synaptic vesicles and the output. This hypothetical concomitant inhibition by the CaM and the cAMP on the CNGL could represent a coincidence detector.

However, the results obtained from the CaM knockdown (CaM-RNAi) combined to either the forskolin or the IBMX or *dnc*-RNAi (Fig. 4*C−E*), which all yield a clear dissociated effect between the CCB and the lobes, could suggest a second alternative—for instance, the implication of intermediate partners. Indeed, the effect of the CaM knockdown in the CCB, which seems to be compensated solely by the direct stimulation of the *rut*-AC (forskolin), is consistent with the canonical model involving CaM directly in the *rut* stimulation. However, the unexpected striking decreased Ca^2+^-response in the ML due to the increase of the cAMP (IBMX or *dnc*-RNAi) combined with CaM knockdown, suggesting that another partner, hypothetically coregulated by cAMP (directly or through PKA) and/or CaM (directly or through CaMKII and/or CASK), could play a role in the ML regulation by inhibiting the Ca^2+^-response when cAMP is increased in absence of the CaM. Interestingly, the Ca^2+^-response of the forskolin in the CaM-RNAi (Fig. 4*C*) resembles the effect of the forskolin, G_αs_, and 8-Br-cAMP in the *cngl*-RNAi context (Fig. 6*F*,*H*,*J*), suggesting that CNGL could be a putative target. Ca^2+^/calmodulin modulation of different CNGs has also been already reported in other systems as olfactory and visual systems (Trudeau and Zagotta, 2003). Therefore, CASK and/or CaMKII are good candidates for these putative intermediate partners (see Fig. 8 for a schematic model) since both of them have been reported to be involved in learning and memory formation (Malik et al., 2013), as well as in calcium signaling in *Drosophila* larvae (Gillespie and Hodge, 2013).

The differential Ca^2+^-response between the CCB and the lobes makes calmodulin a new and interesting candidate for alternative AC activation and so potentially in relation to the coincidence detector hypothesis. However, the L&M effect of the knockdown of calmodulin, specifically within the MBs, would first have to be determined.

## Synthesis

The decision was a result of the Reviewing Editor Muriel Thoby-Brisson and the peer reviewers coming together and discussing their recommendations until a consensus was reached. A fact-based synthesis statement explaining their decision and outlining what is needed to prepare a revision is listed below. The following reviewer agreed to reveal his identity: James Hodge

The proposed paper aims at identifying some components of the cAMP pathway that might be importantly involved in calcium signaling in the mushroom body, a structure that plays a critical role in learning and memory in flies. The study nicely combined in vivo functional imaging (using bioluminescence imaging), genetic manipulations of flies and pharmacological treatments. Using nicotine as a stimulus, the authors show that the Ca2+ response is proportional to the level of cAMP. Then through several genetic manipulations they suggest that in addition to a PKA-dependent effect, a PKA-independent effect through the cyclic nucleotide-gated- channel is also activated. Moreover, there is a differential and regionalized regulation of adenylate cyclase between the calyx/cell bodies and the lobes. Their final conclusion is that cAMP modulates nicotine-induced response via both PKA-dependent and PKA-independent mechanisms. These results provide novel data contributing to a better understand of how cAMP modulates olfactory processing in the mushroom bodies, the learning and memory center of flies.

While the two reviewers agree on the fact that the paper would be suitable for publication in eNeuro, they however consider that significant revisions are required. Indeed the impact of the paper would be improved if some additional experiments would be performed.

1- The reviewers suggest a first series of experiments demonstrating that the CNG-dependent pathway is indeed independent to the PKA pathway. This is stated in the text and the title but not really established. For instance the authors would need to show that (a) the PKA-dependent modulation of calcium response does not depend on CNG channels (for example, whether mC* still increases calcium response in cng-RNAi flies); and (b) the CNG-dependent modulation of calcium response does not depend on PKA activation. The authors are strongly encouraged to perform these experiments. If not they need to strongly justify why not, refocus the paper away from these claims and rewrite the title and abstract.

2-The authors should consider the possibility of using well characterised uas-rut, uas-dnc overexpression lines to show what happens when they bi-directionally change these molecules. These would also be useful for the epistasis experiments with dual manipulation of dnc and rut e.g. can one rescue the other.

3-The use of characterized loss of function (LOF) alleles when possible (eg none lethal, if available) for CMG, CaM, Galpha s would also strengthen the paper. The problem being the RNAi lines is that they were not verified e.g. by qPCR for knockdown of target genes. So if there was no effect on a response it could be because the RNAi line did not knock down the gene e.g. false negative and if it did have a response it could be an off target effect. Confirmation with a chromosomal LOF increases one's confidence. But not in the case of dnc-RNAi not phenocopying dnc1 in figure 3. Can you rescue dnc1 phenotype with adult uas-dnc expression?

For points 3 and 4 the authors may want to do some of these experiments (if not all) and controls or they need to provide strong justification why they have not done them and change the text acccordingly when necessary.

4-Can the rutabaga concidence detector be incorporated into the model eg CaM and Gas causing a superadditive conincident increase in cAMP and therefore Ca. This is the established view of the field. If not then discuss in more detail the rational for why not.

5- Also it appears that important editing and proof reading are required in order to render the paper publishable. Some grammar and typos are listed below:

line (l) Drosophila and all gene names, RNAi, etc italics thoroughout and references

l37 fruitfly italics melanogaster

l45 straight brackets round dnc

l50 remove to

l52 add such after invertebrates

l53 remove of

l58-62 sentence needs to be broken up and rewritten. Also this sentence seems to imply that fluorescence microscopy has some intrinsic weakness for imaging large areas, which is obviously not true (widefield fluorescence microscopy is mostly equivalent to bioluminescence imaging in this regard). People usually focus on smaller structures because it allows for better spatial resolution.

l69 add the after recorded

l70 (using mutants and/or targeted RNAi)

l80 room temperature

l101-103 comment is confusing are you referring to unpublished data? in which case remove or show

l116 remove a

l117 explain coelenterazine, it is not a commonly used term

l143 a 10mM

l144-8 italics Dros

l145 a 13

147 no space between 200 uM throughout

153 signal script dev

155 t-test

156 p in italics

169 justify use of Benjamini-Hochberg test

172 like as

182 remove the (should be noted nAchR are poorly characterised in MB and there are probably multiple forms)

185 natural=endogeneous

189 lobes (ref)

190 and as

195 visualisation of the entire

213 allowed spatio-temporal separation of both of

221 description of lobes needs to be moved earlier in text to the first point lobe data is referred to

226 dendritic branches

232 very

233 remove thes

add results were / different additional

remove from another source

remove have been add which were recorded and then shown to share the same characteristics as

240 in modulation of the MB Ca response

246 decrease in activity

276 Gsα should be Gαs in a number of places

284-5 and throughout Forskolin not Forskoline

304-5 reorder sentence to make clearer

323 to nor like

353: 'though' should be 'through'.

360 negative or positive reinforcement

363 in association with nAchR, make clear this is not actually known but is assumed

368 has not

375-376: 'Fig. 4D' & 'Fig. 4E' should be 'Fig. 4G, H' & 'Fig. 4I, J'?

382 and so in different organisms" not sure what you mean?

389 use to instead of at also "of controls"

400 remove here

402 effects are

405 improve title

422 albeit to a weaker . with not for

449 italics

533 no reference in text to figure 8

539 largely unknown

567-571 poor english rewrite

637 CaMKII

859 brain is bathed

863 emission was

867 explain medial response

877 to the

890 space RNAi (

Fig. 1G: 'Calyx' should be 'Cell bodies', and vice versa, according to the areas marked in Fig. 1H.

8Br-cAMP labeled as '8Br-cAMPc' in Table 1, and labeled as '8Br-AMPc' in Fig. 3 & Fig. 7.

Fig. 7: What do the 'Control', 'CNGs', 'cAMP' rows mean?

Fig. 7: 'cng-RNAi' should be 'cngc-RNAi'?
